# Whole genome sequencing reveals the emergence of a *Pseudomonas aeruginosa* shared strain sub-lineage among patients treated within a single cystic fibrosis centre

**DOI:** 10.1186/s12864-018-5018-x

**Published:** 2018-08-30

**Authors:** Bryan A. Wee, Anna S. Tai, Laura J. Sherrard, Nouri L. Ben Zakour, Kirt R. Hanks, Timothy J. Kidd, Kay A. Ramsay, Iain Lamont, David M. Whiley, Scott C. Bell, Scott A. Beatson

**Affiliations:** 1 Present Address: Usher Institute of Population Health Sciences & Informatics, University of Edinburgh, Edinburgh, United Kingdom; 2 Present Address: Department of Biochemistry, University of Otago, Dunedin, New Zealand; 3 Adult Cystic Fibrosis Centre, Department of Thoracic Medicine, The Prince Charles Hospital, Brisbane, QLD Australia; 4 Present Address: Department of Respiratory Medicine, Western Australia Adult Cystic Fibrosis Centre, Sir Charles Gairdner Hospital, Nedlands, WA Australia; 5 Present Address: School of Pharmacy, Queen’s University Belfast, Belfast, United Kingdom; 6 Centre for Experimental Medicine, Queen’s University Belfast, Belfast, UK; 7 Child Health Research Centre, The University of Queensland, Brisbane, QLD Australia; 8 Department of Biochemistry, University of Otago, Dunedin, New Zealand; 9 Faculty of Medicine, UQ Centre for Clinical Research, The University of Queensland, Brisbane, QLD Australia; 10 Microbiology Department, Pathology Queensland Central Laboratory, Brisbane, QLD Australia; 11 Australian Infectious Diseases Research Centre, The University of Queensland, Brisbane, QLD Australia; 12 Australian Centre for Ecogenomics, The University of Queensland, Brisbane, QLD Australia

**Keywords:** Whole genome sequencing, Cystic fibrosis, *Pseudomonas aeruginosa*, Chronic lung infections, Evolution, AUST-02

## Abstract

**Background:**

Chronic lung infections caused by *Pseudomonas aeruginosa* are a significant cause of morbidity and mortality in people with cystic fibrosis (CF). Shared *P. aeruginosa* strains, that can be transmitted between patients, are of concern and in Australia the AUST-02 shared strain is predominant in individuals attending CF centres in Queensland and Western Australia. M3L7 is a multidrug resistant sub-type of AUST-02 that was recently identified in a Queensland CF centre and was shown to be associated with poorer clinical outcomes. The main aim of this study was to resolve the relationship of the emergent M3L7 sub-type within the AUST-02 group of strains using whole genome sequencing.

**Results:**

A whole genome core phylogeny of 63 isolates indicated that M3L7 is a monophyletic sub-lineage within the context of the broader AUST-02 group. Relatively short branch lengths connected all of the M3L7 isolates. A phylogeny based on nucleotide polymorphisms present across the genome showed that the chronological estimation of the most recent common ancestor was around 2001 (± 3 years). SNP differences between sequential non-hypermutator M3L7 isolates collected 3–4 years apart from five patients suggested both continuous infection of the same strain and cross-infection of some M3L7 variants between patients. The majority of polymorphisms that were characteristic of M3L7 (i.e. acquired after divergence from all other AUST-02 isolates sequenced) were found to produce non-synonymous mutations in virulence and antibiotic resistance genes.

**Conclusions:**

M3L7 has recently diverged from a common ancestor, indicating descent from a single carrier at a CF treatment centre in Australia. Both adaptation to the lung and transmission of M3L7 between adults attending this centre may have contributed to its rapid dissemination. Further genomic investigations are required on multiple intra-sample isolates of this sub-type to decipher potential mechanisms which facilitates its epidemiological success.

**Electronic supplementary material:**

The online version of this article (10.1186/s12864-018-5018-x) contains supplementary material, which is available to authorized users.

## Background

Cystic fibrosis (CF) is the most common recessively lethal inherited disease in people of European ancestry. The majority of mortality and morbidity in people with CF is caused by chronic lung infection with *Pseudomonas aeruginosa* [1]. During chronic infections *P. aeruginosa* adapts to the CF airway microenvironment, which promotes multiple phenotypic and genotypic changes including enhanced resistance to antibiotics, excessive exopolysaccharide production, auxotrophy, auxotrophic metabolism, hypermutability and the loss of motility [2–8]. This evolution strategy has been found to occur in parallel in different *P. aeruginosa* strains, suggesting that these pathoadaptive modifications are important in the transition from an opportunistic pathogen to a specialised pathogen of diseased human lungs [8].

Person-to-person transmission of airway-adapted *P. aeruginosa* strains has also been reported with the acquisition of some “shared strains” (also referred to as “epidemic” or “transmissible” strains) correlated with adverse clinical outcomes [9–13]. AUST-02 (Sequence Type [ST] 775) is a prevalent shared strain in CF centres around Australia and is the dominant strain in Queensland infecting approximately 40% of patients infected with *P. aeruginosa* [10, 14, 15]. A recent study further demonstrated that ongoing evolution within this clonally successful AUST-02 strain has led to the emergence and dissemination of a sub-type within a treatment centre (Brisbane, Queensland) which infected approximately 5% of patients [16]. This sub-type could be distinguished from all other AUST-02 sub-types by a unique *mexZ* (*mexZ-3,* M3) and *lasR* (*lasR*-7, L7) genotype and therefore, was designated M3L7 [16]. The M3L7 sub-type is of particular clinical importance given that it is associated with enhanced multidrug resistance, transmissibility, greater intravenous antibiotic and hospitalisation requirements, and a higher 3-year risk of death/lung transplantation, than other AUST-02 subtypes [16]. Whole genome sequencing (WGS) of 11 longitudinal M3L7 isolates from a single patient revealed the within-host diversity of M3L7 during and after antibiotic treatment of an acute pulmonary exacerbation in 2014 [17], but the M3L7 sub-type has yet to be characterised at the population level.

The aim of this study was to use WGS to reconstruct the population structure and resolve the relationship of the M3L7 sub-type within the AUST-02 group of strains. The analyses revealed that the M3L7 sub-type has recently diverged from a common ancestor, suggesting descent from a single founder within a rapidly growing CF centre population. Genetic mutations exclusive to the M3L7 sub-type were identified and may have aided adaptation to the CF airway microenvironment prior to its dissemination.

## Methods

### Selection of bacterial isolates

One to three *P. aeruginosa* isolates were cultured from single sputum specimens (collected annually in 2007, 2008 and 2011) from adults with CF attending The Prince Charles Hospital as per the Australian Clonal *P. aeruginosa* in Cystic Fibrosis (ACPinCF) study protocol [10]. In brief, sputum samples were prospectively collected and when *P. aeruginosa* was identified, three colonies representing different morphotypes from each specimen were randomly selected. Cross-sectional molecular surveillance for the M3L7 sub-type was conducted in *P. aeruginosa* isolates collected from study participants at two time-points (2007–2008 and 2011). The M3L7 sub-type was detected in 28/509 (5.5%) *P. aeruginosa* isolates collected from 13/170 (7.6%) patients in 2007–2008 and in 21/519 (4.0%) *P. aeruginosa* isolates from 11/173 (6.4%) patients in 2011 [16]. Twenty-five of these M3L7 isolates (named AUS934 to AUS958) were selected from 19 patients (patients 24 to 42; Additional file 1: Table S1) for further WGS as described below.

Of the 13 patients identified with M3L7 in 2007–2008, six patients had no follow-up isolates available (five underwent lung transplantation and one moved interstate by 2011; Patients 24, 32, 33, 34, 35 and 36). Of the seven patients who had M3L7 infection in 2007–2008 and had samples collected in 2011, five remained infected with M3L7 in 2011 (Patients 28, 30, 31, 38 and 41), one (Patient 37) no longer had the M3L7 sub-type detected, while another (Patient 39) tested positive by the M3 allele-specific PCR, but the *lasR* sequence could not be determined because of suboptimal sequence quality (this isolate, AUS947, was subsequently confirmed as M3L7 by WGS in this study). A further five patients (Patients 25, 26, 27, 40 and 42) acquired M3L7 in 2011 (they were infected with other strains in 2007) and were identified as incident cases. Finally, one patient (Patient 29) infected with M3L7 in 2011 had no previous strain-typing data available [16].

One M3L7 isolate was randomly selected per patient at each time-point (2007–2008 and 2011) for WGS and comprised: i) 13 M3L7 isolates from 13 patients in 2007–2008; ii) six M3L7 isolates from six patients with persistent M3L7 infection in 2011; iii) five M3L7 isolates from five incident cases in 2011; and iv) one M3L7 isolate from a patient with no previous *P. aeruginosa* strain typing data collected in 2011. One further *P. aeruginosa* isolate (AUS970) from a patient (patient 43; Additional file 1: Table S1) in 2007 which contained the M3 *mex*Z allele and a non-L7 *lasR* genotype was included as an outgroup. These patients had chronic infection with *P. aeruginosa* prior to 2007 [18, 19].

### Whole genome sequencing

Preparation of genomic DNA for WGS was undertaken using the UltraClean® Microbial DNA Isolation Kit as described previously [17]. Library preparation (Truseq), qPCR (TapeStation, Agilent Genomics) and WGS using the Illumina HiSeq 2500 platform with 100 bp paired-end read chemistry were carried out by the Australian Genome Research Facility, Melbourne, Australia.

In order to reconstruct the M3L7 population structure, a further 37 AUST-02 genomes that were previously sequenced (as part of an ongoing AUST-02 population genetic diversity study (Hanks et al., unpublished)) and a further 11 M3L7 genomes that were previously sequenced (as part of a longitudinal study of patient 37 isolates [17]) were included in the analyses (Additional file 1: Table S1).

### Genome mapping and assembly

Reads were taxonomically assigned with Kraken (v0.10.4) to check for contamination and trimmed using Nesoni clip (v0.128) to filter out adapter sequences and low-quality regions [20, 21]. Reads were mapped to the *P. aeruginosa* PAO1 reference genome (NC_002516) using SHRiMP as implemented in Nesoni (v0.128) [20, 22, 23]. The PAO1 genome was chosen as a reference due to its high quality, and expert-curated annotation [23]. SNPs and small insertions or deletions (indels) shorter than the read length were called using Nesoni.

Genomes were assembled using Velvet (v1.2.10) and VelvetOptimiser (v2.2.5) [24, 25]. Assembled contigs were reordered against PAO1 using Mauve (v2.4.0) and annotated with Prokka (v1.10) [26, 27]. Gene annotations from PAO1 were used as the primary reference. M3L7 and M3L1 draft genome assemblies ranged in total size from 6.15 to 6.25 Mb (mean = 6.24 Mb) with N50 values between 226 Kb and 413 Kb (mean = 319Kb).

### Phylogenetic analysis

An alignment of 30,811 core SNPs obtained from mapping against PAO1 was used to reconstruct the phylogeny of the 63 AUST-02 genome sequences. RAxML (v8.1.15) was used to estimate the Maximum Likelihood tree with the rapid bootstrap analysis option (−f a) and GTRGAMMA model of nucleotide substitution with a correction for ascertainment bias (−m ASC_GTRGAMMA --asc-corr lewis) [28]. A resolved phylogeny of all available M3L7 genome sequences (including the 25 generated in this study, AUS22 (Hanks et al., unpublished) and the 11 isolates from patient 37 in 2014 [17]) was constructed from a SNP matrix of 2573 SNPs with the same settings as above. Phylogenetic trees were viewed and explored using Dendroscope (v3.4.1) and FigTree (v1.4.2) [29, 30]. Minimum spanning trees using the same SNP matrix were generated using the goeBURST Full MST algorithm in Phyloviz using [31].

### BEAST analysis

To determine the emergence of the M3L7 sub-type, Bayesian inference of the evolutionary rates was conducted using BEAST 1.8.2 [32]. As input a set of 183 SNPs specific for M3L7 was used, excluding two hypermutator isolates (AUS937 and AUS938) and the AUS970 outgroup (non-M3L7 isolate). Regions of clustered SNPs, where at least three SNPs were found within 10 bp of each other, were also removed. Among the different combinations of the molecular clock model (strict and constant relaxed lognormal), substitution model (HKY, GTR) and population size change (coalescent constant and exponential growth) models, the preferred combination of parameters selected based on stepping stone sampling was strict molecular clock, HKY substitution model with four discrete gamma-distributed rate categories, and exponential population size change. Markov Chain Monte Carlo generations were run in triplicate for 50 million steps, sampling every 5000 steps, to ensure convergence and an ESS value > 200 for all parameters. Replicate runs were combined using LogCombiner with a 10% burn-in and maximum credibility trees reporting mean values were created using TreeAnnotator.

### Comparative genomic analyses

Comparative genomic analyses were performed using Parsnp, Gingr, BRIG (BLAST Ring Image Generator), Roary (v3.4.2), ACT (Artemis Comparison Tool) and BLAST [33–36]. Large genomic differences were investigated using PHAST (PHAge Search Tool) and Roary [35, 37]. The effect of amino acid substitutions (functionally important or no change) were predicted in silico using PROVEAN (Protein Variation Effect Analyzer) [38]. ABRicate was used to determine acquired resistance gene content by comparing against the ResFinder and CARD database [39]. Multi locus sequence typing (MLST) was performed using the ariba tool and made use of the PubMLST database [40, 41].

## Results and discussion

### M3L7 is a distinct sub-lineage of the AUST-02 shared strain

**Fig. 1:**
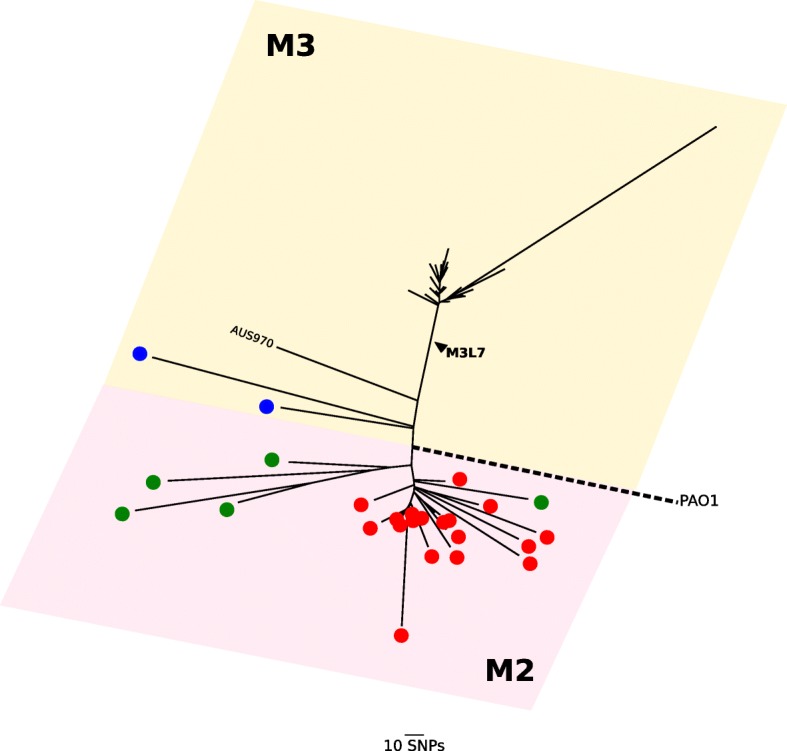
Radial phylogeny of the AUST-02 genomes. The relationship of the M3L7 sub-lineage to other sequenced AUST-02 genomes from patients attending CF centres in Brisbane/Queensland (Red), Perth/Western Australia (Green) and Sydney/New South Wales (Blue: AUS853, AUS854) is shown. The M3 outgroup (AUS970, M3L1) is indicated. The major clades (M2 and M3) are represented by pink and yellow shaded boxes and are defined by different *mexZ* alleles (M2, A38T; M3, T12N). The scale bar represents 10 nucleotide substitutions. Phylogeny was reconstructed estimated from an alignment of 30,811 core genome SNPs (relative to PAO1) using RAxML

On the basis of the whole-genome core phylogeny, the 63 AUST-02 isolates form two major discrete lineages (clades), M2 (*n* = 34) and M3 (*n* = 29), consistent with their possession of *mexZ-2* (codon substitution, A38T) or *mexZ-3* (codon substitution, T12N) alleles, respectively (Fig. 1 and Additional file 2: Figure S1). The M3L7 isolates (*n* = 26; including 25 isolates sequenced here and a previously sequenced AUST-02 genome, AUS22 [Patient 32; 2007]) form a monophyletic sub-lineage of AUST-02 within the M3 clade that has diverged from all other AUST-02 isolates sequenced to date (Fig. 1). Three isolates (AUS853 [Patient 45; 2007]; AUS854 [Patient 44; 2007]; AUS970 [Patient 43; 2007]) within the M3 clade form deep-branching relationships at the base of the lineage and do not harbour the *lasR-7* allele (L7: 1 bp deletion, 438delG) that defines the M3L7 sub-type. Of the available AUST-02 sequences, isolate AUS970 [Patient 43; 2007], sequenced in this study, represented the AUST-02 genome that was most closely related to the M3L7 sub-lineage. In addition to the *mexZ*-*3* allele (M3), AUS970 carried a wild-type *lasR-1* (L1) allele (therefore named M3L1). All 63 isolates are ST-775 according to in silico MLST.

### M3L7 diverged recently from other AUST-02 shared strains

**Fig. 2:**
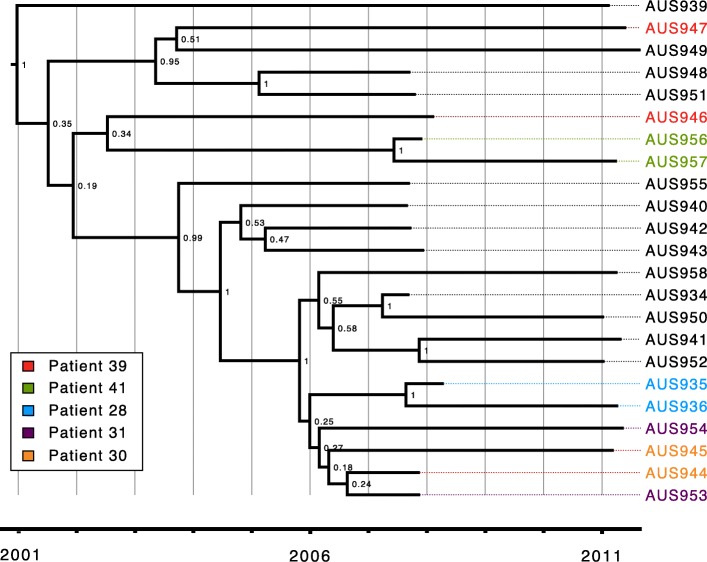
Time-calibrated phylogeny of the M3L7 sub-lineage. Ancestral reconstruction was performed using BEAST 1.8.2 based on a 183 bp non-recombinant SNPs alignment for the 23 non-hypermutator M3L7 strains (sequenced in the current study) isolated between 2007 and 2011, with HKY substitution-, strict clock-, and exponential population tree- models preferred. Posterior probability support is indicated for each node. Paired samples from a patient are coloured according to the legend depicted on the bottom left corner. The x-axis represents the years between 2001 and 2011

The M3L7 sub-lineage expanded recently following a long period of divergence from the M2 sub-lineage according to the relatively short branch lengths connecting the M3L7 isolates and their relative distance from the root (Fig. 1). Using BEAST analysis (Fig. 2), we estimated that the most recent common ancestor (MRCA) of M3L7 emerged around 2001 (± 3 years). This is approximately 6 years prior to the first isolation of an M3L7 sub-type (2007) in people with CF in Brisbane (Additional file 3: Figure S2) [16]. Of note, this time period also corresponds with a relatively high annual increase (approximately 10–15%) in the adult CF population at The Prince Charles Hospital (Additional file 3: Figure S2). This situation, combined with limited capacity to segregate all patients, particularly when admitted to an inpatient ward, may have contributed to shared-strain infections.

The phylogenetic relatedness of M3L7 isolates collected from multiple unrelated patients (including Patients 30, 31, 36, 37, 39, 40, 41) (Additional file 2; Figure S1), also supports the hypothesis of cross-infection. Examination of hospital admission data showed these patients had overlapping periods of hospitalisation to the same inpatient ward at a time when M3L7 was first detected in 2007 (Additional file 4: Table S2). As highlighted previously [16], molecular surveillance showed a striking absence of M3L7 amongst *P. aeruginosa* isolates collected from patients admitted to other wards during the same period. This is emphasised by the lack of concurrent methicillin-resistant *Staphylococcus aureus* or *Burkholderia cepacia* complex infection in those infected with M3L7, which is most likely due to patients with the former two infections receiving treatment in alternate inpatient wards. Baseline sputum samples of four patients with M3L7 infection, included in this study, had additional pathogens identified: *Achromobacter xylosoxidans* (*n* = 3); methicillin-susceptible *Staphylococcus aureus* (*n* = 2); *Mycobacterium abscessus* (*n* = 1). In the year prior to recruitment, a range of co-pathogens were also identified (Additional file 5: Table S3).

The admission data, combined with WGS data, raises the possibility of cross-infection of M3L7 within the inpatient setting. Taken together, these results support a single founder scenario in which a carrier of the M3L7 sub-type acted as a donor within a CF centre with subsequent rapid dissemination in the resident CF population. Alternatively, dissemination of the M3L7 sub-type may have arisen through exposure to common environmental sources; though this is unlikely as earlier surveys of both hospital and natural environmental sites have failed to reveal any common sources of shared CF strains [42–44].

Notably, both sub-lineages of AUST-02 are prevalent in Queensland, suggesting that their MRCA originated in this state; however, we cannot rule out independent introductions of M3L7 and M2 founders into the local CF population from outside the same geographical region (state).

### Resolving the within-patient relationships of M3L7 provides evidence for both continuous infection and person-to-person transmission

To investigate genomic evidence for continuous infection within patients and person-to-person transmission, we examined the pattern of SNP differences between isolates in more detail. As described previously, the dataset included six pairs of M3L7 isolates (Additional file 2: Figure S1) collected from people with CF at two time-points in 2007 or 2008 and in 2011 [16].

A pair of M3L7 isolates (AUS937 and AUS938) from patient 38 had accumulated a much higher number of SNPs compared to other M3L7 isolates, as indicated by their relatively longer branches in the phylogeny (Additional file 2: Figure S1). Further analysis revealed that these two isolates had acquired independent non-synonymous mutations within the *mutS* gene (AUS937, L341P; AUS938, 1 bp deletion [1076delC]), encoding a DNA mismatch repair protein [45], and were predicted to be deleterious to protein function based on an in silico analysis [38]. Mutations in *mutS* are associated with hypermutation, which frequently occurs during chronic *P. aeruginosa* infection of the CF airway [46, 47]. Notably, hypermutators were also identified and characterised in longitudinal isolates collected in 2014 from the person known as patient 37 in the present study [17]. The 2007 M3L7 isolate (AUS951) is closest to AUS961 from the 2014 M3L7 isolates (Additional file 6: Figure S3), with 14 core SNP differences according to a minimal spanning tree of pairwise distances (Additional file 7: Figure S4). In contrast, the two 2014 hypermutator isolates from patient 37 that are closest to AUS951 (AUS965 and AUS966), differ from the 2007 isolate by 335 SNPs or 582 SNPs, respectively. This observation reveals the relative impact of hypermutation on a within-host population over a 7-year time span (Additional file 7: Figure S4), consistent with the previous report of a ~ 40-fold higher mutation rate amongst hypermutable *P. aeruginosa* in long-term CF infections [47].

The minimal spanning tree reveals a maximum of 74 core SNPs separating the most divergent, non-hypermutator isolates, (AUS941 [Patient 27; 2011] and AUS948 [Patient 40; 2011]) isolates (Additional file 7: Figure S4). The closest sequential within-patient isolates (AUS956 and AUS957 from patient 41) differed by only two core SNPs demonstrating a remarkably high degree of genome stability over a 4-year period (Additional file 7: Figure S4). Within-patient isolates from patients 41 (AUS956 and AUS957) and 28 (AUS935 and AUS936) also grouped together with high bootstrap support on the ML phylogeny, which is consistent with continuous M3L7 infection across the two sampling time-points (Additional file 6: Figure S3 and Additional file 7: Figure S4). Our analyses also revealed that a very close relationship existed between the early and late isolates from patients 30 (AUS944 and AUS945, 9 SNPs) and 31 (AUS953 and AUS954, 13 SNPs), which could also be due to continuous infection of the same strain (Additional file 6: Figure S4). Although these four isolates are grouped together with high bootstrap support (Additional file 6: Figure S3), pairwise SNP comparisons reveals only two core genome SNP differences between the 2007 isolates from patients 30 and 31 (AUS944 and AUS953, respectively) (Additional file 7: Figure S4). Notably, the hypothesis of cross-infection between these two patients (or from a common source) is supported by the finding that both patients were admitted to the same inpatient ward, at the same time, approximately 6 months prior to their first positive M3L7 culture (Additional file 4: Table S2).

Based on the Maximum-Likelihood phylogeny, the isolates from patient 39 do not cluster closely (Additional file 6: Figure S3). In fact, AUS947 [patient 39; 2011] is most closely related to AUS961 [patient 37: 2014] and is more closely related to AUS951 [patient 37; 2007] than the earlier 2007 isolate from patient 39 (AUS946) (Additional file 7: Figure S4). This indicates the possibility of multiple M3L7 cross-infection events as has been suggested for other shared strains [48], which may occur via the airborne route or during socialisation between patients [49, 50]. Pairwise SNP comparisons between M3L7 isolates also shows a possible transmission pathway with isolates AUS946 [patient 39; 2007] and AUS944 [patient 30, 2008] being the most likely source of cross-infections (Additional file 7: Figure S4).

AUS22 (an M3L7 isolate sequenced as part of a different study) and AUS943 were both isolated from patient 32 just 6 days apart in 2007. However, these two isolates were more closely related to isolates from other patients than to each other (Additional file 6: Figure S3). Given that only one isolate per sputum sample was analysed, it is unclear if this represents co-existence of multiple related sub-lineages within the chronically infected lungs as we and others have previously shown [5, 7, 17, 47, 48, 51] or if this is also suggestive of direct or indirect cross-infection between those patients. These possibilities are not mutually exclusive.

When analysed together with 11 previously published isolates isolated from a single individual (Patient 37 in this study) during an episode of acute pulmonary exacerbation over 3 months [17], M3L7 isolates from patient 36 (AUS948; 2007) and patient 40 (AUS949; 2011) were found to cluster with the the non-hypermutator isolates from Patient 37 (Additional file 6: Figure S3 and Additional file 7: Figure S4). Interestingly, both hypermutator isolates in patient 38 are more closely related to AUS951 [patient 37; 2007] than to each other, suggesting transmission via a route common to patients 36, 37 and 40, followed by the independent acquisition of *mutS* mutations. These findings highlight that short-term within-patient diversity of shared strains during chronic infection needs to be considered in light of the *P. aeruginosa* lung diversity of the local CF population as a whole.

The resolution of WGS data of multiple isolates from individual patients within samples and longitudinally, combined with social interaction data will enable future studies to fully characterise long-term within-patient M3L7 diversity and evolution and distinguish between continuous infection or recent acquisition of M3L7 variants amongst the CF population [14].

### M3L7 is characterised by an accumulation of non-synonymous mutations in critical pathways

Clonal lineages are expected to accumulate mutations that enable adaptation of the bacterium to a specific environmental niche of a human host [8]. A total of 44 shared SNPs and nine shared indels were acquired after divergence of the M3L7 sub-lineage from all other AUST-02 isolates of the M2 and M3 clades. Thirty-five SNPs were non-synonymous (80%), resulting in a change of the amino acid sequence including two premature stop codons (Additional file 8: Table S4). Four indels produced in-frame mutations, whilst five indels caused a shift in the reading frame (Additional file 8: Table S4). The full list of SNPs and indels found across M3L7 isolates can be found in (Additional file 9: Table S5).

**Fig. 3:**
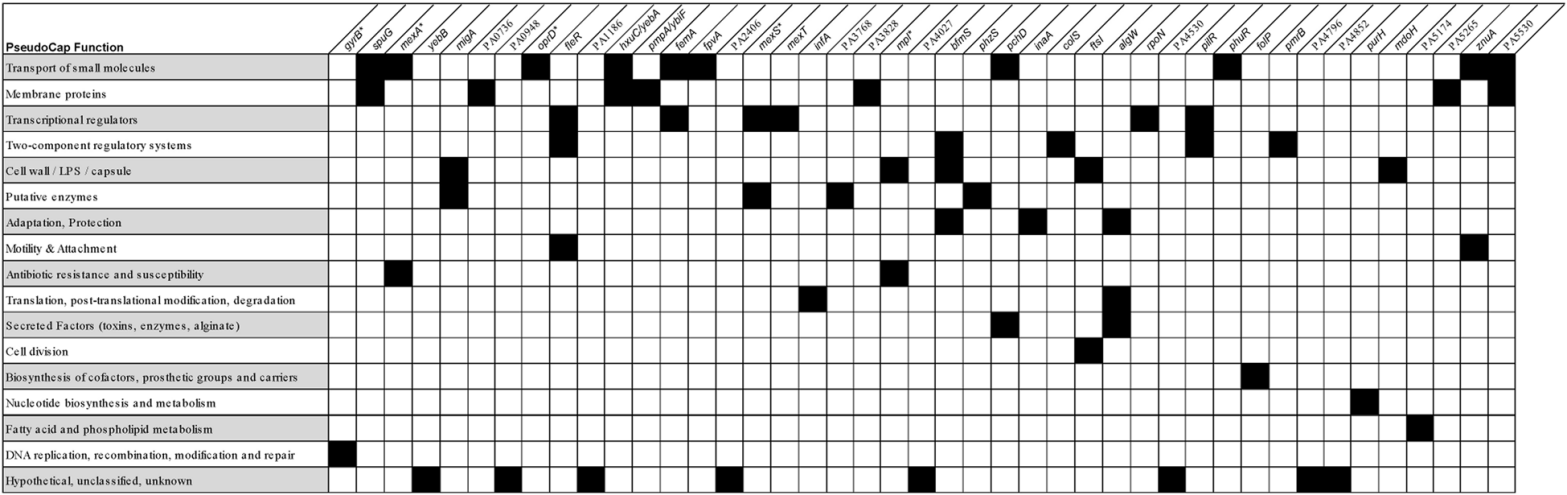
Genes (*n* = 43) with non-synonymous SNPs and indels that characterise the M3L7 sub-lineage. The black squares indicate the PseudoCAP function of the gene. See Additional file 8: Table S4 for detailed information of the specific mutations. *Candidate pathoadaptive genes identified previously [8]

Genes containing non-synonymous SNPs and indels exclusive to the M3L7 sub-lineage (*n* = 43) were subsequently categorised according to PseudoCAP (*P. aeruginosa* community annotation project) functions (Fig. 3) [52]. Fifteen genes were annotated with at least two functional categories and nine genes were part of regulatory networks (Fig. 3), including key global regulators (e.g. *rpoN*, *mexT*), which might impact multiple processes [53]. Approximately 50% of the non-synonymous mutations occurred in genes that encode proteins associated with virulence (e.g. PilR involved in surface attachment and twitching motility [54, 55]; MigA involved in swarming [56]; ZnuA in zinc homeostasis [57]; PchD for iron acquisition [58]) or antibiotic resistance mechanisms (e.g. OprD: carbapenem resistance [59]; GyrB: fluoroquinolone resistance [60]; FtsI, Mpl: β-lactam resistance [61, 62]; PmrB, ColS: polymyxin resistance [63, 64]; MexA, MexS, MexT: multi-drug efflux pumps [65–68]). The mutations found in genes correlated with antibiotic resistance were also identified in M3L7 isolates collected in 2014 [17] and might help explain the increased resistance to anti-pseudomonal antibiotics, including meropenem, ceftazidime, ticarcillin-clavulanate, aztreonam, ciprofloxacin and colistin, compared to other AUST-02 subtypes, as described previously [16]. We did not find any acquired antibiotic resistance genes in the M3 clade isolates. Altogether this analysis suggests that the M3L7 sub-lineage is characterised by mutations in genes that might have aided adaptation to the CF airways prior to dissemination in the CF population [4, 8, 47, 69].

### Genomic variation due to large deletions

The draft genomes that comprise the M3L7 sub-lineage ranged in size from 6.15 to 6.25 Mbp, whereas the M3L1 isolate (AUS970) genome was substantially smaller (6.05 Mbp). All genomes within the M3 clade had an average GC content of 66.5%. Genome reduction is a typical evolutionary process that occurs during adaptation [70]; therefore, large genomic variations (> 10 kb) were compared between the AUST-02 isolates.

Major differences in genome size were not due to horizontal gene transfer but attributed to several deletion events, summarised in Table 1. For example, one M3L7 isolate (AUS947; Patient 39; 2011) lost a large 93 Kbp region encoding a number of virulence genes including *exoY* (a T3SS secreted toxin), a three-part hydrogen cyanide biosynthesis operon, *hcnABC* and part of the *cupA1-A5* chaperone usher fimbrae operon (Table 1). Deletions of varying sizes encompassing *exoY* and *hcnABC* (of the same region) were also missing in three other isolates within the M3 clade (AUS853; [Patient 45; 2007]; AUS854 [Patient 44; 2007]; AUS970 [Patient 43; 2007]) suggesting that there was a selective pressure to lose the functionality of genes encoded at this position.

**Table 1:** Comparison of deletion events larger than 10 Kb in the AUST-02 genomes

Size (Kbp)	PAO1 locus tags	Features		Genome (strain/clade; patient ID; collection year)	
		Gene	Function	Absent	Present
50–93	PA2165-PA2217	• *exoY* • *hcnABC* • *cupA1-A5*	T3SS secreted toxin Hydrogen cyanide biosynthesis operon Chaperone usher fimbrae operon	• AUS947 (M3L7; Patient 39; 2011) • AUS970 (M3L1; Patient 43; 2007) • AUS853 (M3 clade; Patient 45; 2007) • AUS854 (M3 clade; Patient 44; 2007)	• All other AUST-02 isolates
40	PA3619-PA3620	• Adjacent to *mutS*	Putative prophage	• AUS958 (M3L7; Patent 29; 2011) • AUS970 (M3L1; Patient 43; 2007) • AUS853 (M3 clade; Patient 45; 2007) • AUS854 (M3 clade; Patient 44; 2007)	• All other M3L7 isolates • Some Perth isolates^a^ • AUS24 (M2 clade; Patient 16; 2007)
14	PA1914- PA1923	• PA1914 • *nrdD* • PA1922	Encodes halovibrin Pathogenesis related factor TonB-dependent receptor	• All M3L7 isolates	• All other AUST-02 isolates
12	PA2229- PA2237	• *pslABCDEFG*	Biofilm formation	• All M3L7 isolates • AUS853 (M3 clade; Patient 45; 2007)	• All other AUST-02 isolates

^a^AUS15 (M2 clade; Patient 18; 2008); AUS874 (M2 clade; Patient 22; 2008); AUS17 (M2 clade; Patient 17; 2008); AUS876 (M2 clade; Patient 21; 2008); AUS877 (M2 clade; Patient 20; 2008)

A 40 Kbp putative prophage was present in five AUST-02 isolates from Perth (AUS15 [Patient 18; 2008]; AUS874 [Patient 22; 2008]; AUS17 [Patient 17; 2008]; AUS876 [Patient 21; 2008]; AUS877 [Patient 20; 2008]) and one isolate from Brisbane (AUS24; [Patient 16; 2007]) of the M2 clade. This prophage is present in nearly all M3L7 genomes (absent in AUS958 [Patient 29; 2011]) and a previous study also observed the presence of the prophage in 9/11 M3L7 isolates collected in 2014 [17]. Therefore, it is most likely that this prophage was present in the last common ancestor of the M3L7 sub-lineage and was vertically inherited. The prophage is inserted immediately adjacent to the *mutS* gene and a search of the putative prophage sequence using the PhaST webtool predicts a complete prophage that is only 9% identical to the *P. aeruginosa* F10 phage [37]. The prophage is flanked by an 18 bp *att* sequence (TCTCTCAGCACACGCC) that delineates the deletion in AUS958.

## Conclusions

The persistence of the AUST-02 shared strain in the CF population in Australia has led to the emergence of a monophyletic sub-lineage (M3L7) that is distinct from the M2 sub-lineage of AUST-02. Our WGS analysis demonstrated that M3L7 strains are characterised by mutations in genes that are likely to affect antibiotic resistance and virulence phenotypes. The rapid dissemination of this clinically important sub-type is most likely due to a combination of adaptation to the CF airway microenvironment and transmission between people attending the same CF centre. This work provides a framework for future efforts in real-time genomic surveillance to monitor the transmission and pathogenicity of AUST-02 amongst the Australian CF population and to detect newly emergent shared strains. Further genomic investigations are required on multiple intra-sample isolates of this sub-type to decipher potential mechanisms which facilitates its epidemiological success.

## Additional files

Additional file 1: **Table S1.** Details of *Pseudomonas aeruginosa* isolates and genome assembly metrics used in this study. (XLSX 14 kb)

Additional file 2: **Figure S1.** Phylogeny of sequenced AUST-02 strains showing the M3L7 isolates in relation to other AUST-02 isolates within the major M2 and M3 clades. The Maximum-Likelihood phylogenetic tree was estimated from an alignment of 30,811 core genome SNPs using RAxML. > 70% Bootstrap support (*), 100% bootstrap support (**). Scale indicates branch length representing 10 nucleotide substitutions. M2, *mexZ-2* allele (codon substitution, A38T); M3, *mexZ-3* allele (codon substitution, T12N). Pairs of isolates (collected in 2007 or 2008 and 2011) are indicated by colour. Dotted lines indicate branches that have been shortened and are not to scale. Geographic location of CF treatment centre where each isolate was obtained is shown on the right of the tree. (PDF 107 kb)

Additional file 3: **Figure S2.** Growth of the adult CF population at The Prince Charles Hospital between 2001 and 2015. (PDF 64 kb)

Additional file 4: **Table S2.** Overlapping admissions to a hospital ward for patients and first detection of M3L7. (XLSX 10 kb)

Additional file 5: **Table S3.** Details of co-pathogens in people with M3L7/M3L1 infection at time of isolation and in the previous 12 months. (XLSX 11 kb)

Additional file 6: **Figure S3.** A Maximum-Likelihood phylogeny inferred from an alignment of 2573 SNPs showing detailed relationships within the M3L7 clade. The maximum likelihood tree was generated using RAxML with 1000 bootstrap replicates. Branches with * indicate bootstrap support of > 70%. Scale indicates branch length representing 5 nucleotide substitutions. Isolates (*n* = 26) sequenced as part of this study are highlighted in bold font. Isolates that were sequenced as part of other studies are italicised. Isolates originating from the same individual have the same label colour. The year of collection is indicated. The M3L1 (AUS970) outgroup was used to root the tree. M3, *mexZ-3* allele (codon substitution, T12N); L1, *lasR-1* allele (wild-type); L7, *lasR-7* allele (1 bp deletion, 438delG). (PDF 95 kb)

Additional file 7: **Figure S4.** Core SNP based minimum spanning tree depicting the most likely route of transmission. Two isolates (AUS944, AUS946) from this study were predicted to be closest to the source. Edge lengths are not to scale. Details of all SNPs found across M3L7 isolates can be found in Additional file 9: Table S5. Figure generated using the goeBURST Full MST algorithm implemented in Phyloviz [31]. (PDF 63 kb)

Additional file 8: **Table S4.** Position and details of non-synonymous SNPs (*n* = 35) and indels (*n* = 9) that characterise the M3L7 sub-lineage. (XLSX 13 kb)

Additional file 9: **Table S5.** Table of variants (SNPs and indels) that vary across 37 M3L7 genomes and 1 M3L1 (AUS970) genome. Table generated with nesoni nway. Only positions with a consensus determined for all strains (−-require-all yes) are shown. Only substitution SNPs were used to build phylogeny. (XLSX 1466 kb)

## Abbreviations

AUST-02: Australian shared strain/ epidemic strain/ transmissible strain
CF: Cystic fibrosis
M3L1: Sub-lineage of the AUST-02 defined by mexZ (mexZ-3, M3) and lasR (lasR-1, L1) alleles
M3L7: Sub-lineage of the AUST-02 defined by mexZ (mexZ-3, M3) and lasR (lasR-7, L7) alleles
ML: Maximum likelihood
MRCA: Most recent common ancestor
PCR: Polymerase chain reaction
SNP: Single nucleotide polymorphism
ST: Sequence type
WGS: Whole genome sequencing
