# Supplementary material for: Whole genome sequencing reveals the emergence of a Pseudomonas aeruginosa shared strain sub-lineage among patients treated within a single cystic fibrosis centre
Source: BMC Genomics. 2018 Aug 30;19:644. doi: 10.1186/s12864-018-5018-x (PMC6117919; doi:10.1186/s12864-018-5018-x)
Supplement: Supplementary file 2 — Figure S1. Phylogeny of sequenced AUST-02 strains showing the M3L7 isolates in relation to other AUST-02 isolates within the major M2 and M3 clades. The Maximum-Likelihood phylogenetic tree was estimated from an alignment of 30,811 core genome SNPs using RAxML. > 70% Bootstrap support (*), 100% bootstrap support (**). Scale indicates branch length representing 10 nucleotide substitutions. M2, mexZ-2 allele (codon substitution, A38T); M3, mexZ-3 allele (codon substitution, T12N). Pairs of isolates (collected in 2007 or 2008 and 2011) are indicated by colour. Dotted lines indicate branches that have been shortened and are not to scale. Geographic location of CF treatment centre where each isolate was obtained is shown on the right of the tree. (PDF 107 kb) [file 12864_2018_5018_MOESM2_ESM.pdf]

Additional File 2

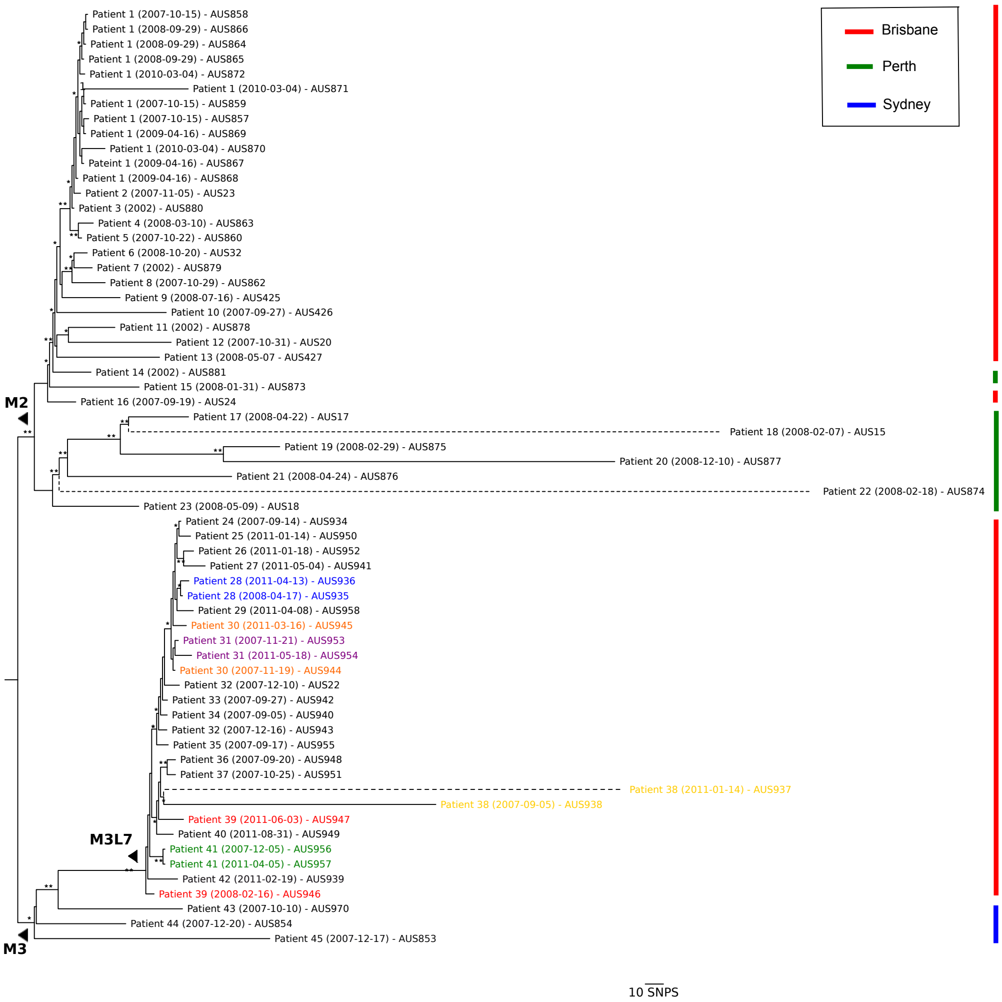

Figure S1. Phylogeny of sequenced AUST-02 strains showing the M3L7 isolates in relation to other AUST-02 isolates within the major M2 and M3 clades. The Maximum-Likelihood phylogenetic tree was estimated from an alignment of 30,811 core genome SNPs using RAxML. >70% Bootstrap support (*), 100% bootstrap support (**). Scale indicates branch length representing 10 nucleotide substitutions. M2, *mexZ-2* allele (codon substitution, A38T); M3, *mexZ-3* allele (codon substitution, T12N). Pairs of isolates (collected in 2007 or 2008 and 2011) are indicated by colour. Dotted lines indicate branches that have been shortened and are not to scale. Geographic location of CF treatment centre where each isolate was obtained is shown on the right of the tree.
