# Supplementary material for: Whole genome sequencing reveals the emergence of a Pseudomonas aeruginosa shared strain sub-lineage among patients treated within a single cystic fibrosis centre
Source: BMC Genomics. 2018 Aug 30;19:644. doi: 10.1186/s12864-018-5018-x (PMC6117919; doi:10.1186/s12864-018-5018-x)
Supplement: Supplementary file 3 — Figure S2. Growth of the adult CF population at The Prince Charles Hospital between 2001 and 2015. (PDF 64 kb) [file 12864_2018_5018_MOESM3_ESM.pdf]

Additional File 3

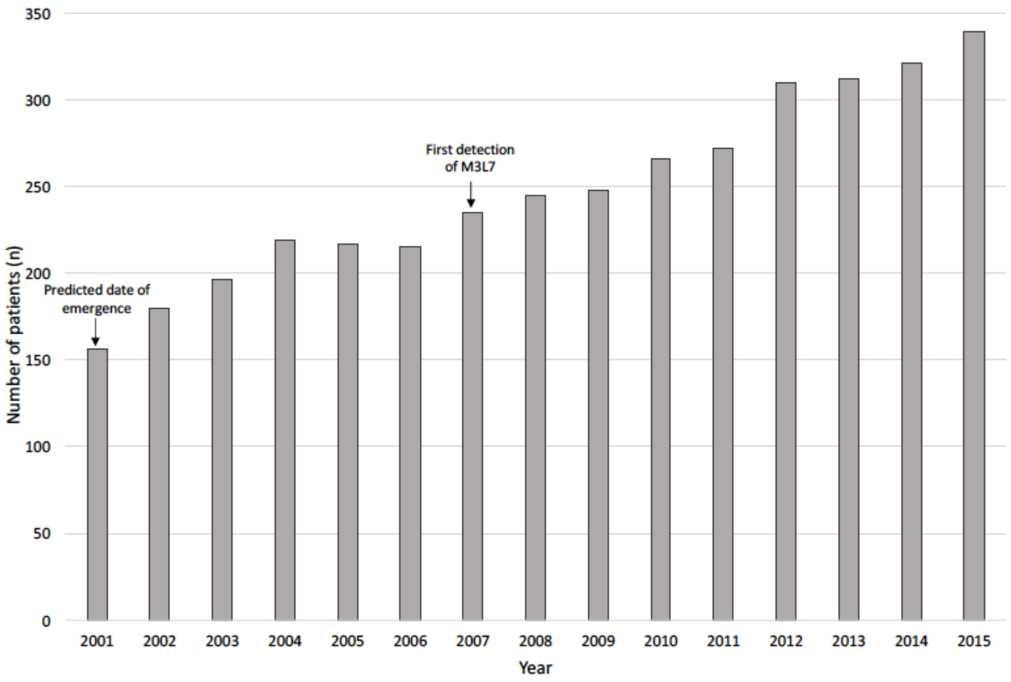

Figure S2. Growth of the adult CF population at The Prince Charles Hospital between 2001 and 2015.
