# Supplementary material for: Whole genome sequencing reveals the emergence of a Pseudomonas aeruginosa shared strain sub-lineage among patients treated within a single cystic fibrosis centre
Source: BMC Genomics. 2018 Aug 30;19:644. doi: 10.1186/s12864-018-5018-x (PMC6117919; doi:10.1186/s12864-018-5018-x)
Supplement: Supplementary file 6 — Figure S3. A Maximum-Likelihood phylogeny inferred from an alignment of 2573 SNPs showing detailed relationships within the M3L7 clade. The maximum likelihood tree was generated using RAxML with 1000 bootstrap replicates. Branches with * indicate bootstrap support of > 70%. Scale indicates branch length representing 5 nucleotide substitutions. Isolates (n = 26) sequenced as part of this study are highlighted in bold font. Isolates that were sequenced as part of other studies are italicised. Isolates originating from the same individual have the same label colour. The year of collection is indicated. The M3L1 (AUS970) outgroup was used to root the tree. M3, mexZ-3 allele (codon substitution, T12N); L1, lasR-1 allele (wild-type); L7, lasR-7 allele (1 bp deletion, 438delG). (PDF 95 kb) [file 12864_2018_5018_MOESM6_ESM.pdf]

Additional File 6

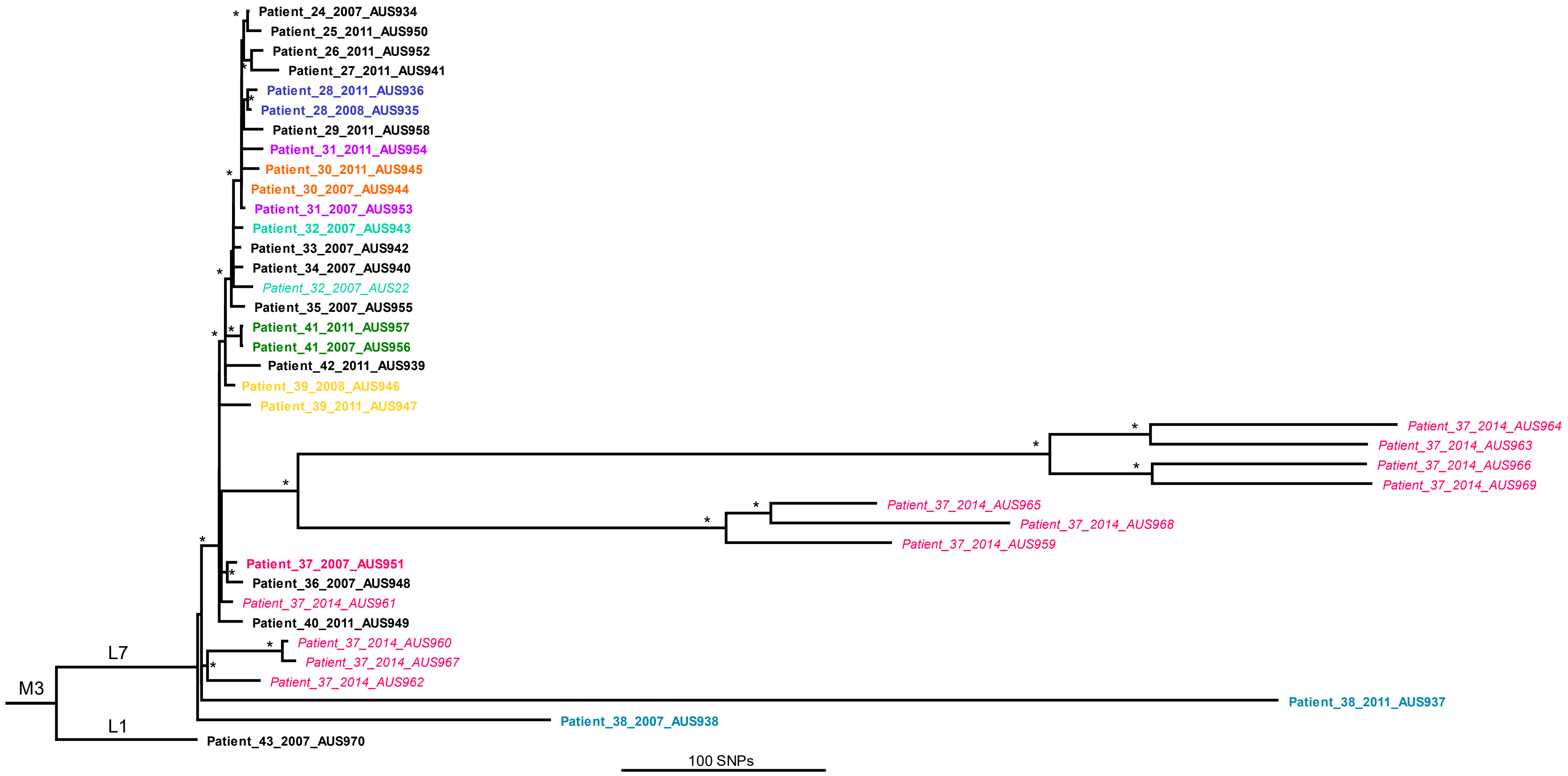

Figure S3. A Maximum-Likelihood phylogeny inferred from an alignment of 2573 SNPs showing detailed relationships within the M3L7 clade. The maximum likelihood tree was generated using RAxML with 1000 bootstrap replicates. Branches with * indicate bootstrap support of >70%. Scale indicates branch length representing 5 nucleotide substitutions. Isolates (n=26) sequenced as part of this study are highlighted in bold font. Isolates that were sequenced as part of other studies are italicised. Isolates originating from the same individual have the same label colour. The year of collection is indicated. The M3L1 (AUS970) outgroup was used to root the tree. M3, *mexZ-3* allele (codon substitution, T12N); L1, *lasR-1* allele (wild-type); L7, *lasR-7* allele (1 bp deletion, 438delG).
