# Supplementary material for: Whole genome sequencing reveals the emergence of a Pseudomonas aeruginosa shared strain sub-lineage among patients treated within a single cystic fibrosis centre
Source: BMC Genomics. 2018 Aug 30;19:644. doi: 10.1186/s12864-018-5018-x (PMC6117919; doi:10.1186/s12864-018-5018-x)
Supplement: Supplementary file 7 — Figure S4. Core SNP based minimum spanning tree depicting the most likely route of transmission. Two isolates (AUS944, AUS946) from this study were predicted to be closest to the source. Edge lengths are not to scale. Details of all SNPs found across M3L7 isolates can be found in Additional file 9: Table S5. Figure generated using the goeBURST Full MST algorithm implemented in Phyloviz [31]. (PDF 63 kb) [file 12864_2018_5018_MOESM7_ESM.pdf]

Additional File 7

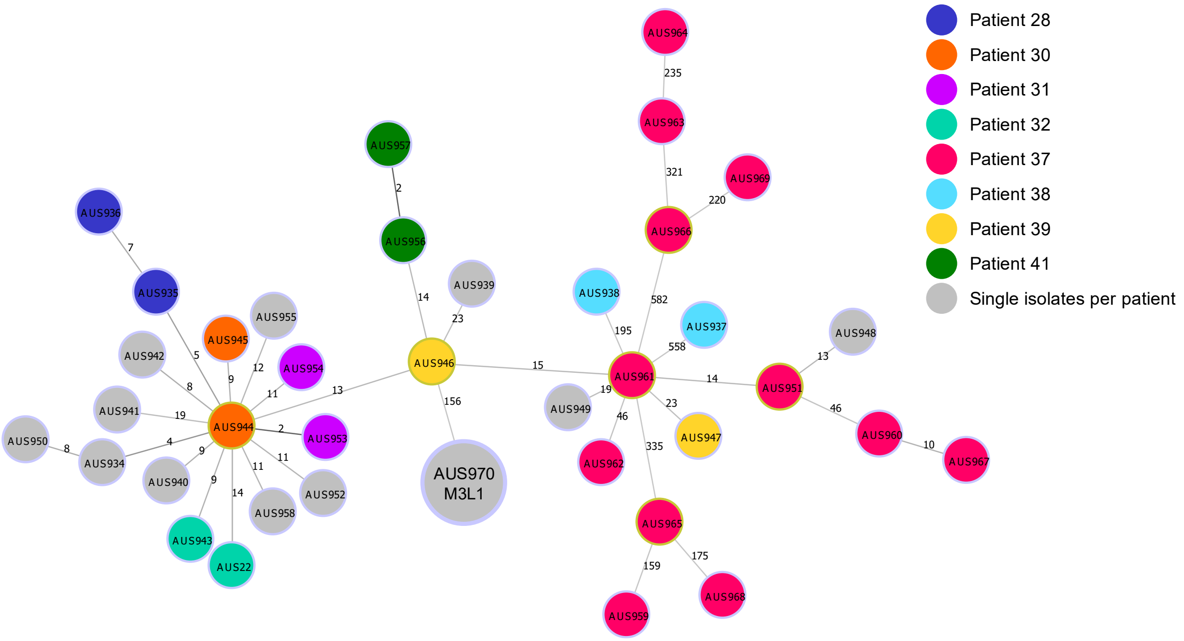

Figure S4. Core SNP based minimum spanning tree depicting the most likely route of transmission. Two isolates (AUS944, AUS946) from this study were predicted to be closest to the source. Edge lengths are not to scale. Details of all SNPs found across M3L7 isolates can be found in Additional File 9: Table S5. Figure generated using the goeBURST Full MST algorithm implemented in Phyloviz
